# Reciprocal regulation of forkhead box C1 and L1 cell adhesion molecule contributes to triple-negative breast cancer progression

**DOI:** 10.1007/s10549-023-07177-7

**Published:** 2024-01-06

**Authors:** Fan Zhang, Yue Xu, Jiediao Lin, Hongchao Pan, Armando E. Giuliano, Xiaojiang Cui, Yukun Cui

**Affiliations:** 1 Oncology Research Laboratory, Cancer Hospital of Shantou University Medical College, Shantou, Guangdong China; 2 Guangdong Provincial Key Laboratory for Breast Cancer Diagnosis and Treatment, Cancer Hospital of Shantou University Medical College, Shantou, Guangdong China; 3 Department of Surgery, Cedars-Sinai Medical Center, Samuel Oschin Comprehensive Cancer Institute, Los Angeles, CA USA

**Keywords:** Triple-negative breast cancer, Forkhead box C1, L1 cell adhesion molecule, Reciprocal regulation, Cell proliferation, Metastasis

## Abstract

**Purpose:**

The potential of targeting forkhead box C1 (FOXC1) as a therapeutic approach for triple-negative breast cancer (TNBC) is promising. However, a comprehensive understanding of FOXC1 regulation, particularly upstream factors, remains elusive. Expression of the L1 cell adhesion molecule (L1CAM), a transmembrane glycoprotein associated with brain metastasis, was observed to be positively associated with FOXC1 transcripts. Thus, this study aims to investigate their relationship in TNBC progression.

**Methods:**

Publicly available FOXC1 and L1CAM transcriptomic data were obtained, and their corresponding proteins were analyzed in four TNBC cell lines. In BT549 cells, FOXC1 and L1CAM were individually silenced, while L1CAM was overexpressed in BT549-shFOXC1, MDA-MB-231, and HCC1937 cells. CCK-8, transwell, and wound healing assays were performed in these cell lines, and immunohistochemical staining was conducted in tumor samples.

**Results:**

A positive correlation between L1CAM and FOXC1 transcripts was observed in publicly available datasets. In BT549 cells, knockdown of FOXC1 led to reduced L1CAM expression at both the transcriptional and protein levels, and conversely, silencing of L1CAM decreased FOXC1 protein levels, but interestingly, FOXC1 transcripts remained largely unaffected. Overexpressing L1CAM resulted in increased FOXC1 protein expression without significant changes in FOXC1 mRNA levels. This trend was also observed in BT549-shFOXC1, MDA-MB-231-L1CAM, and HCC1937-L1CAM cells. Notably, alterations in FOXC1 or L1CAM levels corresponded to changes in cell proliferation, migration, and invasion capacities. Furthermore, a positive correlation between L1CAM and FOXC1 protein expression was detected in human TNBC tumors.

**Conclusion:**

FOXC1 and L1CAM exhibit co-regulation at the protein level, with FOXC1 regulating at the transcriptional level and L1CAM regulating at the post-transcriptional level, and together they positively influence cell proliferation, migration, and invasion in TNBC.

**Supplementary Information:**

The online version contains supplementary material available at 10.1007/s10549-023-07177-7.

## Introduction

In 2020, breast cancer surpassed lung cancer as the most frequently diagnosed malignancy worldwide [1]. Among the distinct subtypes of breast cancer, triple-negative breast cancer (TNBC) accounts for approximately 15%, and is characterized by the absence of estrogen receptor (ER), progesterone receptor (PR), and human epidermal growth factor receptor 2 (HER2) expression [2]. TNBC is associated with a higher risk of visceral metastases, particularly to the lungs and brain [3]. Due to the lack of ER and HER2 expression, endocrine therapy and anti-HER2-targeted therapy are ineffective for TNBC, and patients with TNBC brain metastases have a poor prognosis with an average survival time of less than two years [4]. Current treatment primarily relies on chemotherapy, underscoring the need for additional therapeutic targets for TNBC.

FOXC1 (forkhead box C1) is a member of the FOX family of transcription factors [5]. While it plays a critical role in embryonic development [6], its involvement in tumor progression across various cancers has been demonstrated. Notably, FOXC1 is consistently present in basal-like breast cancer, an intrinsic subtype that comprises the majority of triple-negative tumors [7], and its detrimental role is indicated by its activation of NF-κB signaling [8], induction of epithelial-to-mesenchymal transition [9], promotion of breast cancer stem cell properties [10], and downregulation of ER expression [11]. In breast cancer, FOXC1 expression is also positively associated with brain and lung metastases [12]. We previously have shown that FOXC1 enhances TNBC cell invasion, motility, and lung metastasis, possibly by activating the transcription of chemokine receptor-4 [13]. However, for translational medicine purposes, a comprehensive understanding of FOXC1's upstream and downstream regulators in TNBC necessitates further investigation [5].

L1 cell adhesion molecule (L1CAM), an immunoglobulin (Ig) superfamily member, is a transmembrane glycoprotein [14]. While its primary functions relate to neuronal migration, axon growth, and synapse formation in the brain, L1CAM has been consistently detected in tumors over the past two decades and is implicated in tumor progression [14]. In breast cancer, L1CAM expression is observed in all molecular subtypes but shows a preference for TNBC and is associated with aggressive behavior [15, 16]. L1CAM has been implicated in vascular co-option in brain metastasis [17], although the triggers for L1CAM expression in cancer cells remain incompletely understood [18]. Thus, further elucidation of regulators affecting L1CAM expression in TNBC is needed.

In our study, we analyzed publicly available RNA sequencing data from breast cancer patients, and identified a positive correlation between FOXC1 and L1CAM expression (Pearson correlation coefficient = 0.370, 0.244). Given their shared characteristics in tumor progression, it is plausible that L1CAM may be associated with FOXC1 in TNBC. We observed that (1) silencing FOXC1 reduced L1CAM expression, potentially through transcriptional inactivation; (2) dysregulation of L1CAM resulted in corresponding changes in FOXC1 levels, independent of transcriptional regulation; (3) aberrant expression of either FOXC1 or L1CAM positively influenced TNBC cell proliferation, migration, and invasion in vitro. These results suggest the existence of reciprocal co-regulation between FOXC1 and L1CAM, contributing at least partially to the aggressive behavior of TNBC cells.

## Material and methods

### Public databases

Two patient cohorts, consisting of 1108 and 379 individuals with breast cancer, were examined in this study. The data was sourced from TCGA (The Cancer Genome Atlas, Firehose legacy), and the Metastatic Breast Cancer Project (Provisional, December 2021). These datasets were accessed via the cBioPortal database (https://www.cbioportal.org/, accessed on November 18th, 2022) [19, 20]. To assess gene expression, messenger RNA (mRNA) values were obtained using the RNA-Seq by Expectation–Maximization (RSEM) method, which produced logarithmic values on a base-2 scale. Pearson's correlation analyses were conducted separately on the two cohorts, comprising 960 and 157 patients, respectively. These patients were selected based on having complete information for both L1CAM and FOXC1.

Furthermore, within TCGA, the transcription levels of L1CAM were compared between two groups: high expression (n = 375) and low expression (n = 585) of FOXC1. The division between the groups was determined using the mean value as the cutoff.

### Cell culture 

The SUM149, HCC1937, MDA-MB-231 and BT549 TNBC cell lines were obtained from the ATCC (American Type Culture Collection, Manassas, VA, USA). Cells were cultured in RPMI 1640 (Gibco, Thermo Fisher, Waltham, USA) supplemented with 10% fetal bovine serum (FBS, Biological Industry, Kibbutz Beit HaEmek, Israel).

### Transient transfection

In preparation for transfection, 2 × 10^5^ cells (BT549, MDA-MB-231, or HCC1937) were seeded into 6-well plates and allowed to reach 80–90% confluence within 24 h. For L1CAM knockdown, BT549 cells were transiently transfected with L1CAM siRNA (BT549-siL1CAM) or control scramble siRNA (BT549-siNC) using Lipofectamine 3000 according to the manufacturer’s instructions (Invitrogen, Carlsbad, CA, USA). The siRNA sequences for L1CAM and control scramble were obtained from HIPPOBIO (Shenzhen, China) as follows; si-L1CAM: forward: 5’-GAACGGCAACCUCUACUUUTT-3’ and reverse: 5’-AAAGUAGAGGUUGCCGUUCTT-3’; siNC: 5’-UUCUCCGAACGUGUCACGUTT-3’ and reverse: 5’-ACGUGACACGUUCGGAGAATT-3’. To deplete FOXC1, BT549 cells were infected with FOXC1 shRNA (BT549-shFOXC1) or a scrambled sequence as a control (BT549-shNC), which were cloned into the pLV-hU6-Neongreen construct (SyngenTech, Beijing, China). Cells with reduced FOXC1 levels were selected using 1 μg/mL puromycin. Additionally, to overexpress L1CAM, BT549-shFOXC1, MDA-MB-231, and HCC1937 cells were transfected with full-length human L1CAM mRNA (NM_000425) inserted into the GV358Vector (GeneChem, Shanghai, China) and were selected using Geneticin at concentrations of 400 μg/mL, 1 mg/mL, and 1 mg/mL for BT549-shFOXC1-L1CAM, MDA-MB-231-L1CAM, and HCC1937-L1CAM cells, respectively.

### Wound healing assays

BT549-shFOXC1, BT549-siL1CAM, BT549-shFOXC1-L1CAM, MDA-MB-231-L1CAM, and HCC1937-L1CAM cell lines, along with their corresponding control cells, were plated at a density of 5 × 10^5^ cells per well in 6-well plates. A uniform wound was created in the cell monolayer using a 1 mL plastic pipette tip. After 24 h of incubation, the wound width was examined using a phase-contrast microscope (NIKON; Konan, Tokyo, Japan). At least 3 random fields were photographed, and the closure of the wound distance was measured at 0 and 24 h. Cell migration percentages (%) were calculated using the formula: [(0-h-gap distance - 24-h-gap distance)/0-h-gap distance] × 100. All measurements were performed using ImageJ software.

### Migration and invasion assays

Cell migration and invasion were assessed using Transwell chambers (Corning, NY, USA) with a polycarbonate membrane (8 μm pore diameter). For invasion assays, the polycarbonate membrane was coated with Matrigel (50 μl BD Biosciences, Franklin Lakes, New Jersey, US). Each cell line was suspended in 200 μl of serum-free RPMI 1640 medium at a concentration of 5 × 10^4^ cells and seeded into the upper chamber. The lower chamber was filled with 600 μl of RPMI1640 medium supplemented with 10% FBS. After 24 h, the upper chamber and cells on the upper surface of the membrane were removed. The cells that had migrated to the lower surface of the membrane were stained with 0.1% crystal violet and counted under a Leica microscope at 100 × magnification (DM3000, Wetzlar, Germany). Four randomly selected fields from each sample were used for cell counting. Results were averaged from three replicates, and each experiment was repeated three times.

### Cell proliferation assays

Cell viability was assessed by seeding cells into 96-well plates at a concentration of 2000 cells per well. After four to five hours, the cells were incubated with 20 μl of CCK-8 solution (C0038, Beyotime Biotechnology, China) for 2.5 h in a humidified chamber at 37 °C. Absorbance was then measured at a wavelength of 450 nm using a microplate reader (Multiskan MK3, Thermo Fisher, CA, US). The optical density (OD value) was recorded. Results were based on four technical replicates, and each experiment was repeated three times.

### Real-time polymerase chain reaction

Total RNAs were isolated from cells using TRIzol (cat#15596026), followed by reverse transcription using oligo (dT) priming and Superscript III reverse transcriptase as per the manufacturer’s instructions (TaKaRa, Tokyo, Japan). Real-time PCR was performed using a SYBR Premix kit (TaKaRa, Tokyo, Japan), with β-actin serving as the loading control. The reactions were carried out on a 7300 Real-Time PCR System (Applied Biosystems, Waltham, MA, USA). Primer pairs for the target genes were obtained from Sangon Biotech (Shanghai, China) as follows:

FOXC1 forward, 5’-TCACAGAGGATCGGCTTGAAC-3’,

FOXC1 reverse, 5’-TCCTGCTTTGGGGTTCGATT-3’,

L1CAM forward, 5’-CCCCGAGGAATTGATGGAGC-3’,

L1CAM reverse, 5’-GGTTCTGGTAGGTGACACGG-3’,

β-actin forward, 5’-CATGTACGTTGCTATCCAGGC-3′,

β-actin reverse, 5’-CTCCTTAATGTCACGCACGAT-3’.

### Western blot analyses

Cells were lysed using a cell lysis buffer containing phenylmethylsulfonyl fluoride (Beyotime, Shanghai, China). Proteins (30 μg) from each cell lysate were separated by SDS-PAGE and transferred onto a polyvinylidene difluoride (PVDF) membrane (Millipore, Bedford, MA, USA). The membrane was blocked with bovine serum albumin and incubated overnight at 4 °C with primary antibodies against FOXC1 (1:500, ab223850; Abcam, Cambridge, UK), L1CAM (1:1000, ab270455; Abcam, Cambridge, UK), GAPDH (1:2000, sc-47724; Santa Cruz Biotechnology, Dallas, TX, USA), and β-tubulin (1:3000, sc-5274; Santa Cruz Biotechnology, Dallas, TX, USA) in blocking buffer. After washing with TBST (Tris-buffered saline with 0.2% Tween 20), the blots were incubated with horseradish peroxidase-labeled anti-rabbit (1:10000, ab205718; Abcam, Cambridge, UK) or anti-mouse (1:5000, ab6728; Abcam, Cambridge, UK) secondary antibodies at room temperature for two hours. Following another round of washing with TBST, the signal was detected using chemiluminescence. Protein bands were visualized using an ECL western blotting substrate (cat#32109, Thermo Fisher, USA).

### Patients and tumor specimens

Paraffin-embedded archival pathological specimens from 40 patients diagnosed with TNBC, along with comprehensive clinicopathological data, were obtained. The patients underwent biopsy without preoperative therapy at the Cancer Hospital of Shantou University Medical College between January 2013 and December 2019. Among the cohort, the majority of patients (n = 34) were diagnosed with early stages (I/II), while the remaining cases were at advanced stages (III/IV), except for one case with an unknown stage. The clinical tumor stage (TNM stage) was classified according to the American Joint Committee on Cancer, 6th Edition Cancer Staging Manual (2002). Informed consent was obtained from all participants after providing detailed information and potential consequences. The study involving tumor samples was approved by the medical ethics committee of the Cancer Hospital of Shantou University Medical College (approval number: 2019024).

### Immunohistochemistry

Immunohistochemistry (IHC) was performed to detect FOXC1 and L1CAM in TNBC. Briefly, 4-μm thick tissue sections were fixed in 10% buffered formalin and embedded in paraffin. After deparaffinization and rehydration, endogenous peroxidase activity was blocked with 0.3% hydrogen peroxide. The sections were then autoclaved in citrate buffer (pH 6.0) and incubated with rabbit anti-FOXC1 monoclonal antibody (1:50, ab223850; Abcam, Cambridge, UK) or L1CAM antibody (1:100, ab270455; Abcam, Cambridge, UK). IHC staining was performed using the EnVision antibody complex (anti-mouse/rabbit) method with an Elivision plus Polymer HP (Mouse/Rabbit) IHC Kit (MXB Biotechnologies, Fujian, China) and 3,3’-diaminobenzidine as the chromogen substrate. Scoring for FOXC1 and L1CAM IHC staining was based on a combination of intensity (0, no staining; 1, weak staining; 2, moderate staining; 3, strong staining) and proportion (0, < 5% of tumor cells stained; 1, 5–25% positive cells; 2, 26–50% positive cells; 3, 51–75% positive cells; 4, more than 76% positive cells). Expression was considered positive if the product of multiplication between staining intensity and the proportion of positive cells was > 4. Two pathologists independently assessed the cellular location and intensity of immunostaining in each section.

### Statistical analyses

Statistical analyses were performed using GraphPad Prism 8.0 software (San Diego, CA). Data are expressed as mean ± standard deviation (SD). Comparisons of transcriptional levels, cell viability and cell mobility between constructed cells and their controls were conducted using *t*-tests. For all tests, a value of *P* < 0.05 was considered significant.

## Results

### L1CAM is down-regulated at the transcriptional and protein level after FOXC1 knockdown

**Fig. 1:**
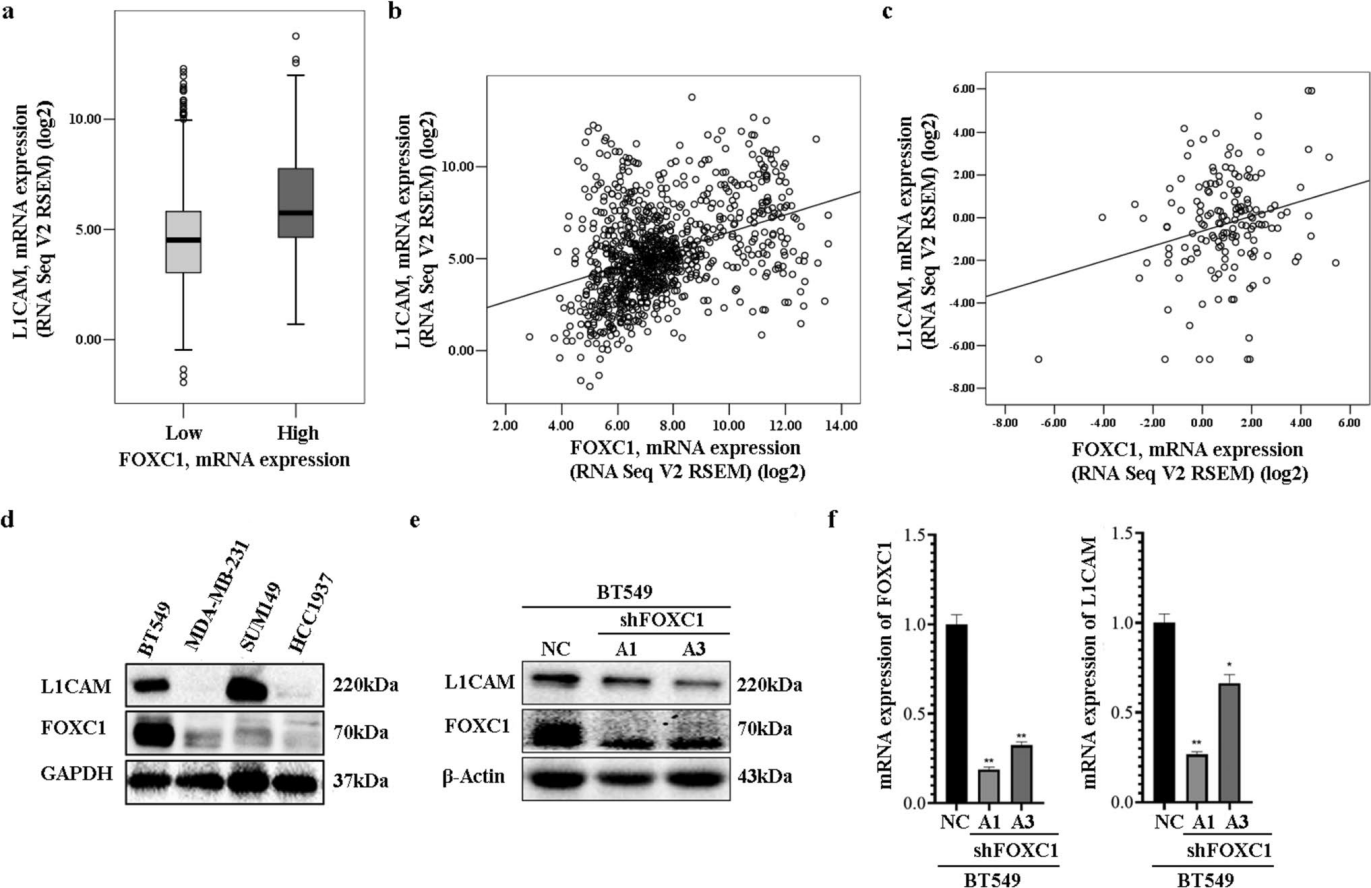
FOXC1 is correlated with L1CAM, and its depletion leads to decreased L1CAM in TNBC cells. **a** Differences in L1CAM mRNA expression were examined between patients with a high (*n* = 375) and low level (*n* = 585) of FOXC1 mRNA. The mean was used for the cutoff value. **b**–**c** Two scatter plots were created to indicate the relation between FOXC1 and L1CAM mRNA expression in two public cohorts: TCGA and the Metastatic Breast Cancer Project, respectively (Pearson’s correlation coefficient *r* = 0.370, *P* < 0.001; *r* = 0.244, *P* = 0.002). **d** FOXC1 and L1CAM proteins were examined in four TNBC cell lines, with GAPDH serving as the loading control. **e** FOXC1 and L1CAM proteins were both down-regulated in BT549-shFOXC1 cells (by two different shFOXC1 sequences: A1 and A3) compared to BT549-shNC. β-Actin served as the loading control. **f** Real-time PCR detected decreased transcriptional levels of FOXC1 and L1CAM in two BT549-shFOXC1 cell clones: BT549-shFOXC1-A1 and BT549-shFOXC1-A3, compared to BT549-shNC. NC: BT549-shFOXC1-NC. A1 and A3: knockdown of FOXC1 with two shRNA sequences: RNA1 and RNA3 in BT549 cells, respectively. *P < 0.05, **P < 0.01, ***P < 0.001

A correlation analysis was performed using publicly available mRNA profiles from two cohorts of breast cancer patients to investigate the relationship between L1CAM and FOXC1. Figure 1a shows that patients with high FOXC1 levels exhibited higher L1CAM expression compared to those with low FOXC1 levels (*P* < 0.001). Furthermore, L1CAM demonstrated a significant positive correlation with FOXC1 (*r* = 0.37, *P* < 0.001, Fig. 1b; *r* = 0.244, *P* = 0.002, Fig. 1c). Subsequently, both L1CAM and FOXC1 proteins were examined in four TNBC cell lines (Fig. 1d). BT549, which exhibited detectable expression of both genes, was primarily used for subsequent experiments. Knockdown of FOXC1 resulted in a considerable decrease in FOXC1 mRNA and protein expression in two BT549-shFOXC1 cell clones (BT549-shFOXC1-A1 and BT549-shFOXC1-A3, Fig. 1e–f). Concurrently, a reduction in L1CAM mRNA and protein levels was also observed (Fig. 1e–f), suggesting that FOXC1 might, to some extent, regulate L1CAM expression at the transcriptional level.

### Down-regulation of L1CAM reduces proliferation, invasion and migration of TNBC cells along with reducing FOXC1 protein levels

**Fig. 2:**
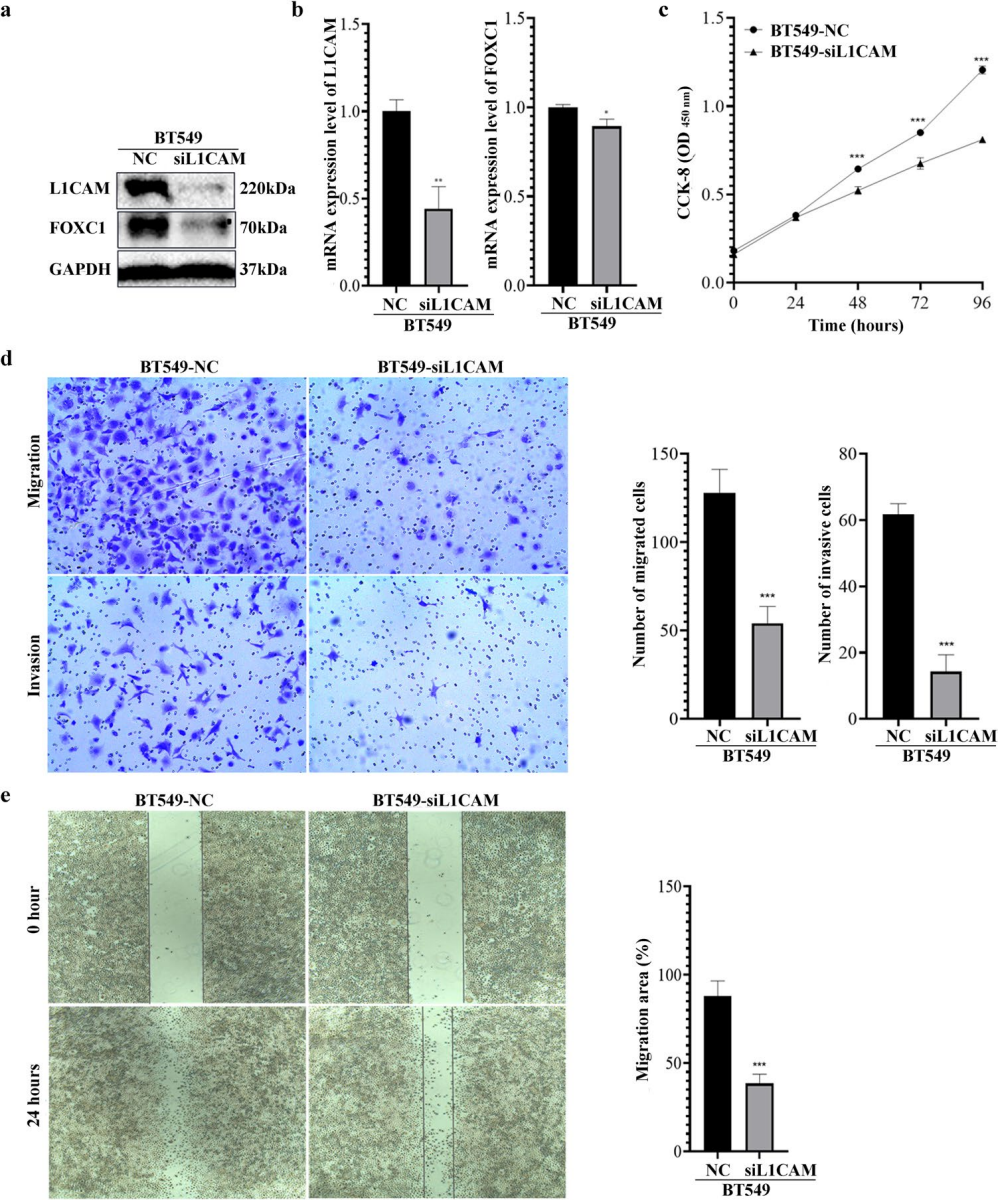
Inhibition of L1CAM suppresses expression of FOXC1 proteins, cell proliferation, invasion and migration. **a** Down regulation of both L1CAM and FOXC1 proteins was detected in BT549-siL1CAM cells. GAPDH served as the loading control. **b** Real-time PCR detected a considerable decrease of L1CAM mRNA, but only a slight decrease of FOXC1 mRNA in BT549-siL1CAM cells. **c** CCK-8 assay showed L1CAM knockdown decreased the capacity of cell proliferation. **d** Cell migration and invasion were impaired after L1CAM knockdown in BT549 cells. **e** L1CAM knockdown suppressed BT549 cell migration in a wound healing assay. NC: negative control, siL1CAM: a small interfering RNA used to knockdown L1CAM mRNA in BT549 cells. *P < 0.05, **P < 0.01, ***P < 0.001

To investigate the role of L1CAM in TNBC cells and its impact on FOXC1, BT549-siL1CAM cells with reduced L1CAM mRNA and protein were generated (Fig. 2a–b). Interestingly, we observed that the knockdown of L1CAM simultaneously decreased FOXC1 protein levels (Fig. 2a). However, only a slight decrease in FOXC1 mRNA levels (0.11-fold decrease, Fig. 2b) was observed compared to controls, suggesting that L1CAM may reciprocally regulate FOXC1 expression through a mechanism independent of transcription.

CCK-8 assays demonstrated that the viability of BT549-siL1CAM cells decreased after 48 (OD values: 0.523 vs. 0.644, *P* < 0.001, Fig. 2c), 72 (0.676 vs. 0.851, *P* < 0.001), and 96 h (0.811 vs. 1.205,* P* < 0.001) compared to parental cells. Transwell assays revealed impaired metastatic capacity in BT549-siL1CAM cells (number of migrated cells: 54 ± 7 vs. 128 ± 13, *P* < 0.001; number of invaded cells: 14 ± 5 vs. 62 ± 3, *P* < 0.001, Fig. 2d). Similarly, wound healing assays confirmed reduced migration of BT549-siL1CAM cells (migration area: 38.5% vs. 88.0%,* P* < 0.001, Fig. 2e). These findings indicate that BT549-siL1CAM cells exhibit decreased cell viability and impaired metastatic ability, potentially associated with the reduction of FOXC1 proteins.

### Overexpression of L1CAM promotes cell invasion and migration through FOXC1

**Fig. 3:**
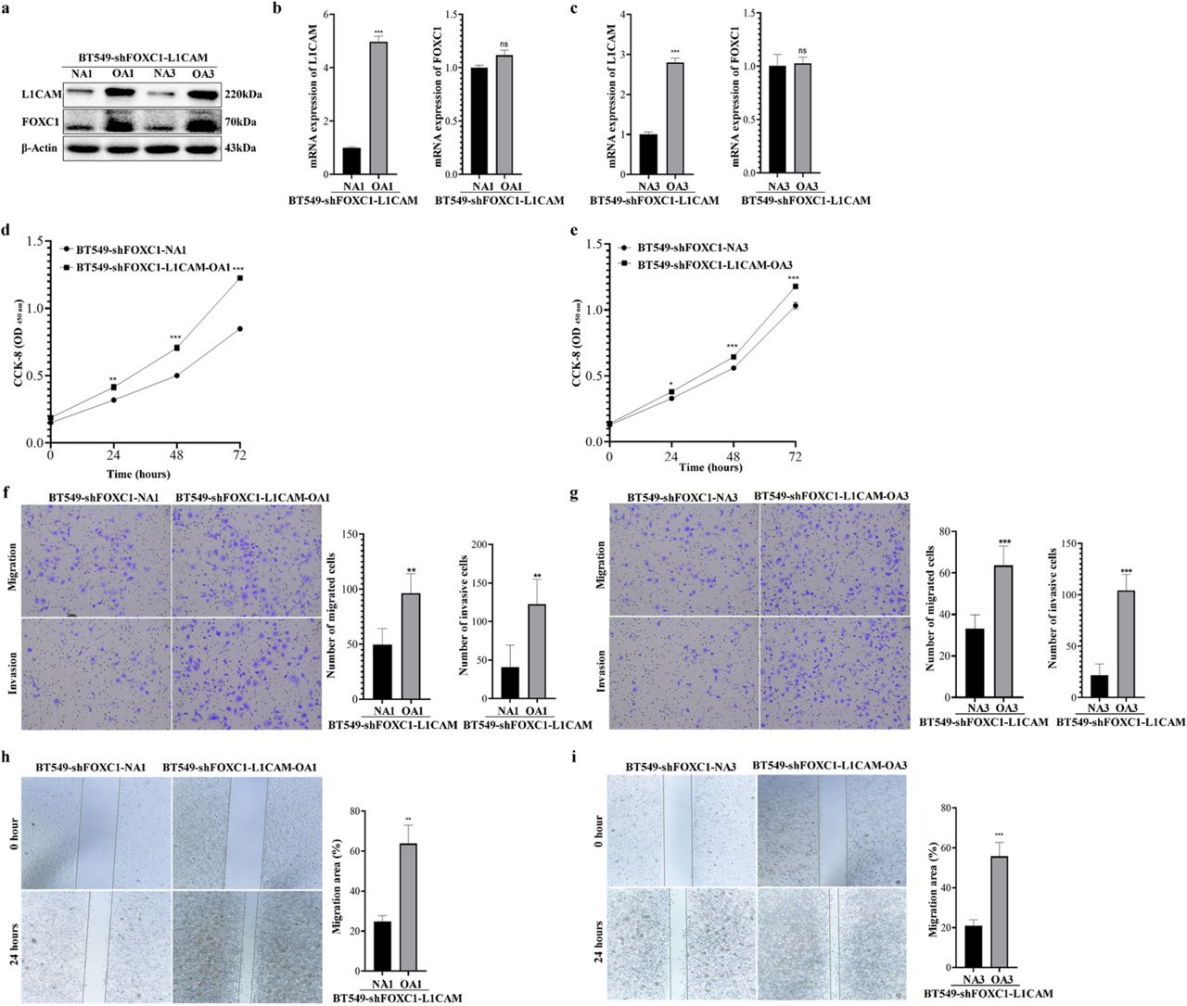
Overexpression of L1CAM induces expression of FOXC1 proteins, cell proliferation, cell invasion and migration. **a** Western blotting showed L1CAM and FOXC1 proteins after overexpression of L1CAM in BT549-shFOXC1-A1 and BT549-shFOXC1-A3, i.e., BT549-shFOXC1-L1CAM-OA1 and BT549-shFOXC1-L1CAM-OA3. Their controls were represented as BT549-shFOXC1-NA1 and BT549-shFOXC1-NA3. β-Actin served as the loading control. **b**–**c** Real-time PCR showed the relative mRNA expression of L1CAM and FOXC1 after overexpressing L1CAM in BT549-shFOXC1 cells. **d**–**e** CCK-8 assays showed the effect of L1CAM overexpression on cell proliferation in BT549-shFOXC1-L1CAM. **f**–**g** Cell migration and invasion were evaluated after overexpressing L1CAM in BT549-shFOXC1 cells. **h–i** Upregulation of L1CAM in BT549-shFOXC1 cells enhanced cell migration in vitro wound healing assays. NA1: BT549-shFOXC1-A1 transfected with empty vector control. OA1: BT549-shFOXC1-A1 cells overexpressing L1CAM. NA3: BT549-shFOXC1-A3 transfected with empty vector control. OA3: BT549-shFOXC1-A3 cells overexpressing L1CAM. *P < 0.05, **P < 0.01, ***P < 0.001

To further investigate the role of L1CAM in TNBC cells and its impact on FOXC1, BT549-shFOXC1 cells were engineered to overexpress L1CAM in two BT549-shFOXC1 cell clones (i.e., BT549-shFOXC1-L1CAM-OA1 and BT549-shFOXC1-L1CAM-OA3), and the corresponding controls were represented as BT549-shFOXC1-NA1 and BT549-shFOXC1-NA3 (Fig. 3a–c). Interestingly, in these cells, FOXC1 protein levels were increased (Fig. 3a), while there was only a negligible change at the mRNA level. CCK-8 assays showed an increase in cell proliferation for BT549-shFOXC1-L1CAM cells compared to controls (e.g., BT549-shFOXC1-L1CAM-OA1 vs. BT549-shFOXC1-NA1: 24 h: 0.414 vs. 0.319, *P* < 0.001; 48 h: 0.707 vs. 0.501, *P* < 0.001; 72 h: 1.225 vs. 0.848, *P* < 0.001, Fig. 3d–e). Transwell assays indicated enhanced migration and invasion capacities (e.g., BT549-shFOXC1-L1CAM-OA1 vs. BT549-shFOXC1-NA1: number of migrated cells: 96 ± 18 vs. 50 ± 15, *P* < 0.001; number of invaded cells: 123 ± 32 vs. 41 ± 29, *P* < 0.001, Fig. 3f–g). Wound healing assays confirmed an increased migratory capacity of BT549-shFOXC1-L1CAM cells compared to controls (Fig. 3h–i). These findings suggest that the restoration of proliferation, invasion, and migration in BT549-shFOXC1-L1CAM cells could be attributed to the upregulation of FOXC1.

Similar results were observed in two other TNBC cell lines, MDA-MB-231-L1CAM and HCC1937-L1CAM, which also overexpressed L1CAM. These cell lines exhibited increased levels of FOXC1 protein but only a moderate increase in FOXC1 mRNA (Online Resource Fig s1,a–b and Fig. S2a–b). CCK-8 assays and wound healing assays demonstrated that L1CAM overexpression in MDA-MB-231 and HCC1937 cells enhanced cell proliferation and migration (Online Resource Fig s1c, S1e, Fig. S2c, S2e), while transwell assays did not show an increased capacity for invasion in MDA-MB-231-L1CAM (Online Resource Fig s1d).

### L1CAM and FOXC1 are correlated at the protein level in human breast cancer

**Fig. 4:**
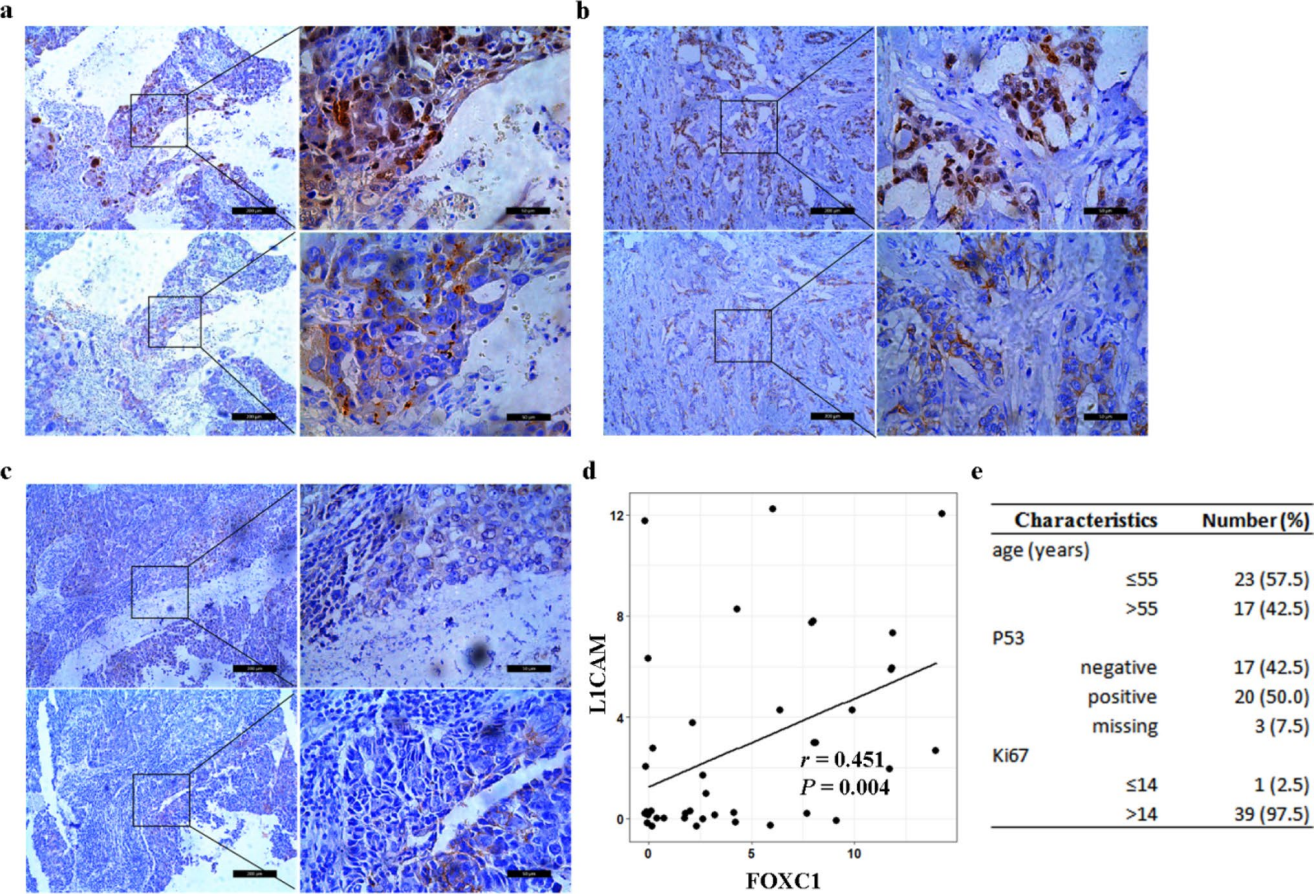
Expression correlation between L1CAM and FOXC1 was detected by IHC. **a-c** Examples for strong, moderate and week IHC staining of FOXC1 (upper left: 100 × ; upper right: 400 ×) and L1CAM (lower left: 100 × ; lower right: 400 ×) in TNBC tissue sections from the same tumor are shown. **d** A scatter plot of FOXC1 IHC scores to L1CAM IHC scores was created (Pearson’s correlation coefficient *r* = 0.451, *P* = 0.004). **e** Some clinical-pathological information of these patients was provided

The relationship between FOXC1 and L1CAM in TNBC was examined by investigating their protein expression levels using IHC in tumor samples from 40 TNBC patients. Representative images of FOXC1 and L1CAM staining are shown in Fig. 4a–c. There was a significant correlation between FOXC1 and L1CAM expression (*r* = 0.451, *P* = 0.004, Fig. 4d). Clinicopathological information of these samples is presented in Fig. 4e. These findings indicate a positive correlation between L1CAM expression and FOXC1 in TNBC tissues.

## Discussion

This study initially observed a decrease in L1CAM expression, both at the protein and transcriptional levels following FOXC1 knockdown. Notably, down- or up-regulating L1CAM levels in turn resulted in decreased or increased expression of FOXC1, primarily at the protein level. These changes were accompanied by impaired or enhanced capacities of proliferation, migration, and invasion of TNBC cells. Collectively, these findings suggest a reciprocal regulation between FOXC1 and L1CAM, which is related to the proliferation, migration, and invasion of TNBC cells.

FOXC1 and L1CAM are known to be present in various types of cancer and have been implicated in cancer development based on in vitro experiments. They are consistently associated with poor prognosis in cancer patients [14, 21], and in the context of breast cancer, both FOXC1 and L1CAM exhibit a preference for triple-negative breast cancer (TNBC) over other subtypes. FOXC1 has been shown to promote distant metastasis to the lung and brain in TNBC [12, 13, 22], while L1CAM is implicated in vascular co-option during brain metastasis [17, 23]. However, a recent immunohistochemistry (IHC) study failed to detect L1CAM expression in a series of thirty resected breast cancer brain metastases [24]. The authors proposed the possibility that L1CAM may no longer be necessary and thus downregulated after the formation of macro-metastases in the brain. Here, we present the hypothesis that the downregulation of L1CAM in brain metastases could be attributed to the silencing of FOXC1. Although FOXC1 was not specifically examined in that study, it is noteworthy that most brain metastases collected in that paper were either ER-positive or HER2-positive tumors, which are less likely to express FOXC1 [24].

FOXC1 and L1CAM may share common mechanisms in breast cancer progression. Despite extensive studies on tumor progression [5], the regulation of FOXC1 remains poorly understood. In this study, we observed a significant change in FOXC1 protein levels without corresponding changes in transcripts, along with a corresponding change in L1CAM expression. Indicated by previous studies, the NF-κB and MAPK signaling pathways may serve as potential mediators between these two genes. NF-κB is particularly prominent in basal-like tumors, in contrast to ER-positive breast cancer [25]. In vitro studies have demonstrated that L1CAM acts as a ligand for integrins in MDA-MB-231 cells, leading to NF-κB activation [26]. Notably, NF-κB can bind to the FOXC1 promoter and initiate transcription [27]. Therefore, NF-κB may facilitate the interaction between L1CAM and FOXC1. Interestingly, we did not observe significant changes in FOXC1 transcripts despite the substantial alteration in FOXC1 protein levels. This suggests that transcriptional regulation may not be the primary mechanism by which L1CAM regulates FOXC1 expression. A recent study highlighted that FOXC1 is regulated by p38 MAPK, with Ser241 and Ser272 identified as critical phosphorylation sites for FOXC1 protein stability, without affecting mRNA levels [28]. Moreover, inhibition of L1CAM expression by L1CAM-specific siRNA suppresses the activation of MAPKs, such as ERK and p38 [29, 30]. Therefore, it is possible that p38 or related pathways contribute to the underlying mechanism by which L1CAM influences FOXC1 expression at the protein level, rather than at the transcriptional level. However, these hypotheses have yet to be confirmed in breast cancer cells, and further investigation is ongoing to explore these possibilities.

In addition to the positive influence of L1CAM on FOXC1, we also observed a reduction in FOXC1 expression leading to decreased L1CAM levels, likely due to transcriptional downregulation. The specific underlying mechanism for this observation remains unclear. Previous studies have indicated that β-catenin is a direct transcriptional target of FOXC1 [31–33], and L1CAM is a target gene of the β-catenin signaling pathway [34]. This suggests that β-catenin may play a role in the link between FOXC1 and L1CAM. However, further investigation is required to determine whether FOXC1 directly or indirectly regulates L1CAM in breast cancer cells.

The upstream regulation of L1CAM and FOXC1 expression has been extensively reviewed [5, 14]. However, a more comprehensive understanding of their regulation in TNBC is still needed to fully explore their potential as therapeutic targets. In this study, we demonstrate the possibility of a reciprocal regulation between FOXC1 and L1CAM, which is involved in the proliferation, migration, and invasion of TNBC cells. One exception is that the cell invasion capacity was not enhanced in MDA-MB-231 cells after L1CAM upregulation as BT549-shFOXC1 and HCC1937 cells did; it could be due to different genetic backgrounds and mutation statuses among these cell lines. While the individual functions of FOXC1 and L1CAM in TNBC have been extensively studied, our focus here is on their interrelation in TNBC cells. To the best of our knowledge, this is the first study to suggest that L1CAM may regulate FOXC1, and vice versa, and that the former may not rely on transcriptional regulation. However, the specific regulatory mechanisms underlying FOXC1 and L1CAM are not yet clear, and we aim to address this in future studies.

### Supplementary Information

Below is the link to the electronic supplementary material.

Supplementary file1 (DOC 2680 KB)

## Data Availability

The public datasets analyzed during the current study are available in cBioPortal, https://www.cbioportal.org/. The data generated during the current study is available from the corresponding author on reasonable request.

## Abbreviations

ER: Estrogen receptor
FOXC1: Forkhead box C1
HER2: Human epidermal growth factor receptor 2
IHC: Immunohistochemistry
L1CAM: L1 cell adhesion molecule
OD: Optical density
PR: Progesterone receptor
RSEM: RNA-Seq by expectation maximization
SD: Standard deviation
TNBC: Triple-negative breast cancer
