# Supplementary material for: Reciprocal regulation of forkhead box C1 and L1 cell adhesion molecule contributes to triple-negative breast cancer progression
Source: Breast Cancer Res Treat. 2024 Jan 6;204(3):465–74. doi: 10.1007/s10549-023-07177-7 (PMC10959774; doi:10.1007/s10549-023-07177-7)

**Supplementary materials**

・Journal: Breast Cancer Research and Treatment

・Title: Reciprocal regulation of forkhead box C1 and L1 cell adhesion molecule contributes to triple-negative breast cancer progression

・Authors’ names: Fan Zhang**^1^**^†^, Yue Xu**^2^**^†^, Jiediao Lin**^2^**, Hongchao Pan**^1^**, Armando E. Giuliano**^3^**, Xiaojiang Cui**^3^**, Yukun Cui**^2*^**,

・Authors’ affiliations:

**^1^**Oncology Research Laboratory, Cancer Hospital of Shantou University Medical College, Shantou 515041, Guangdong, China.

**^2^**Guangdong Provincial Key Laboratory for Breast Cancer Diagnosis and Treatment, Cancer Hospital of Shantou University Medical College, Shantou, 515041, Guangdong, China.

**^3^**Department of Surgery, Samuel Oschin Comprehensive Cancer Institute, Cedars-Sinai Medical Center, Los Angeles, CA, United States.

・Corresponding author: Yukun Cui, Guangdong Provincial Key Laboratory for Breast Cancer Diagnosis and Treatment, Cancer Hospital of Shantou University Medical College, Shantou 515041, Guangdong, China. Email: ykcui@stu.edu.cn; Fax: (86)-0754-88560352.

**List of Supporting Information**:

**Fig S1 Overexpression of L1CAM induces FOXC1 protein expression, cell proliferation and migration, but not invasion of MDA-MB-231 cells**

(**a**) Western blot showing L1CAM and FOXC1 proteins after overexpression of L1CAM in MDA-MB-231 cells. GAPDH served as the loading control. (**b**) Real-time PCR showing the relative mRNA expression of L1CAM and FOXC1 after overexpressing L1CAM in MDA-MB-231 cells. (**c**) CCK-8 assays showing the effect of L1CAM overexpression on cell proliferation in MDA-MB-231 cells. (**d**) Cell migration and invasion were evaluated after overexpressing L1CAM in MDA-MB-231 cells. (**e**) Upregulation of L1CAM in MDA-MB-231 cells enhanced cell migration in vitro wound healing assays. **P*<0.05, ***P*<0.01, ****P*<0.001


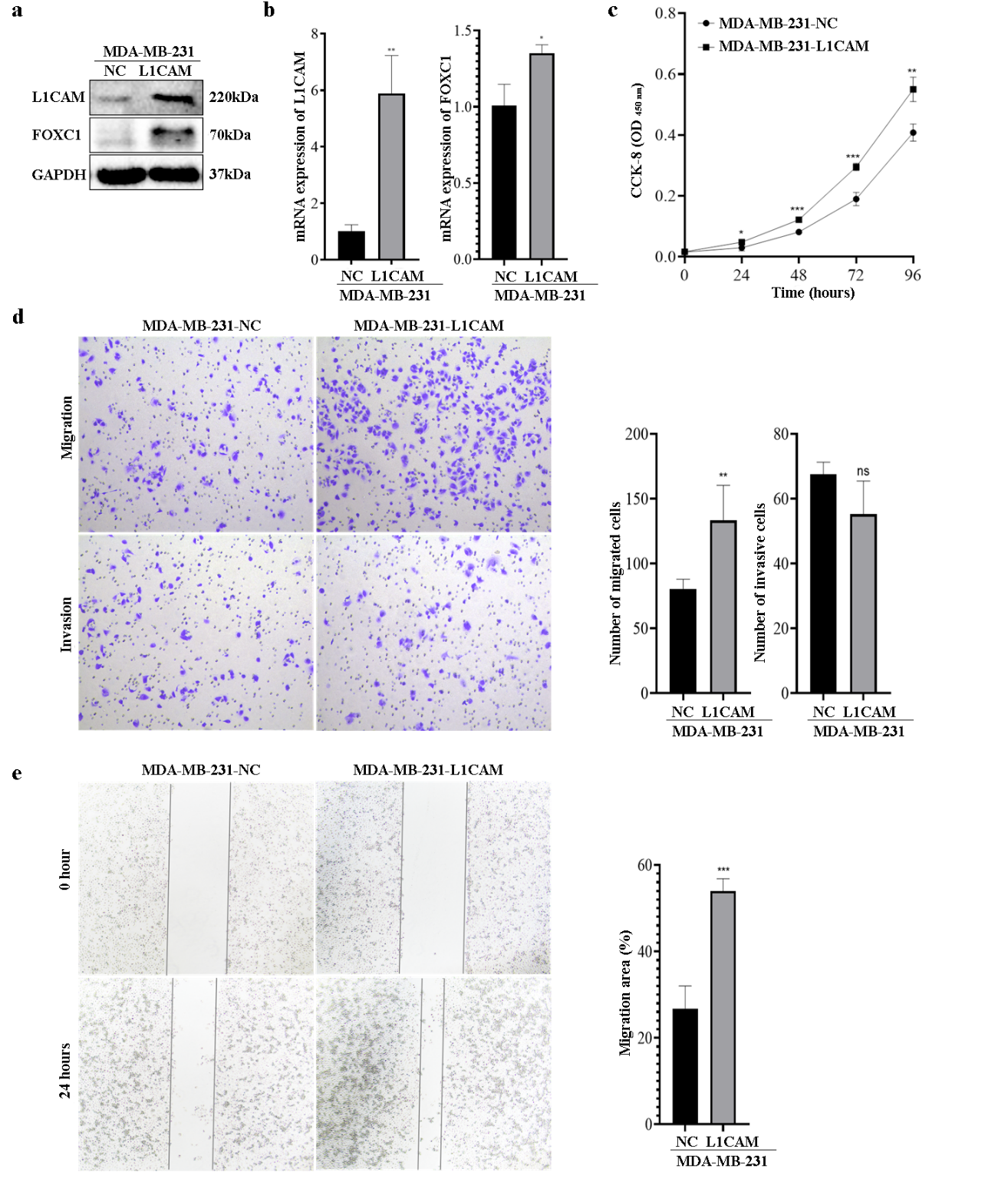


**Fig S2 Overexpression of L1CAM enhances expression of FOXC1 protein, cell proliferation, invasion and migration in HCC1937 cells**

**(**a) Western blot showing L1CAM and FOXC1 proteins after overexpression of L1CAM in HCC1937 cells. GAPDH served as the loading control. (**b**) Real-time PCR showing the relative mRNA expression of L1CAM and FOXC1 after overexpressing L1CAM in HCC1937 cells. (**c**) CCK-8 assays showing the effect of L1CAM overexpression on cell proliferation in HCC1937 cells. (**d**) Cell migration and invasion were evaluated after overexpressing L1CAM in HCC1937 cells. (**e**) Upregulation of L1CAM in HCC1937 cells enhanced cell migration in vitro wound healing assays. **P*<0.05, ***P*<0.01, ****P*<0.001


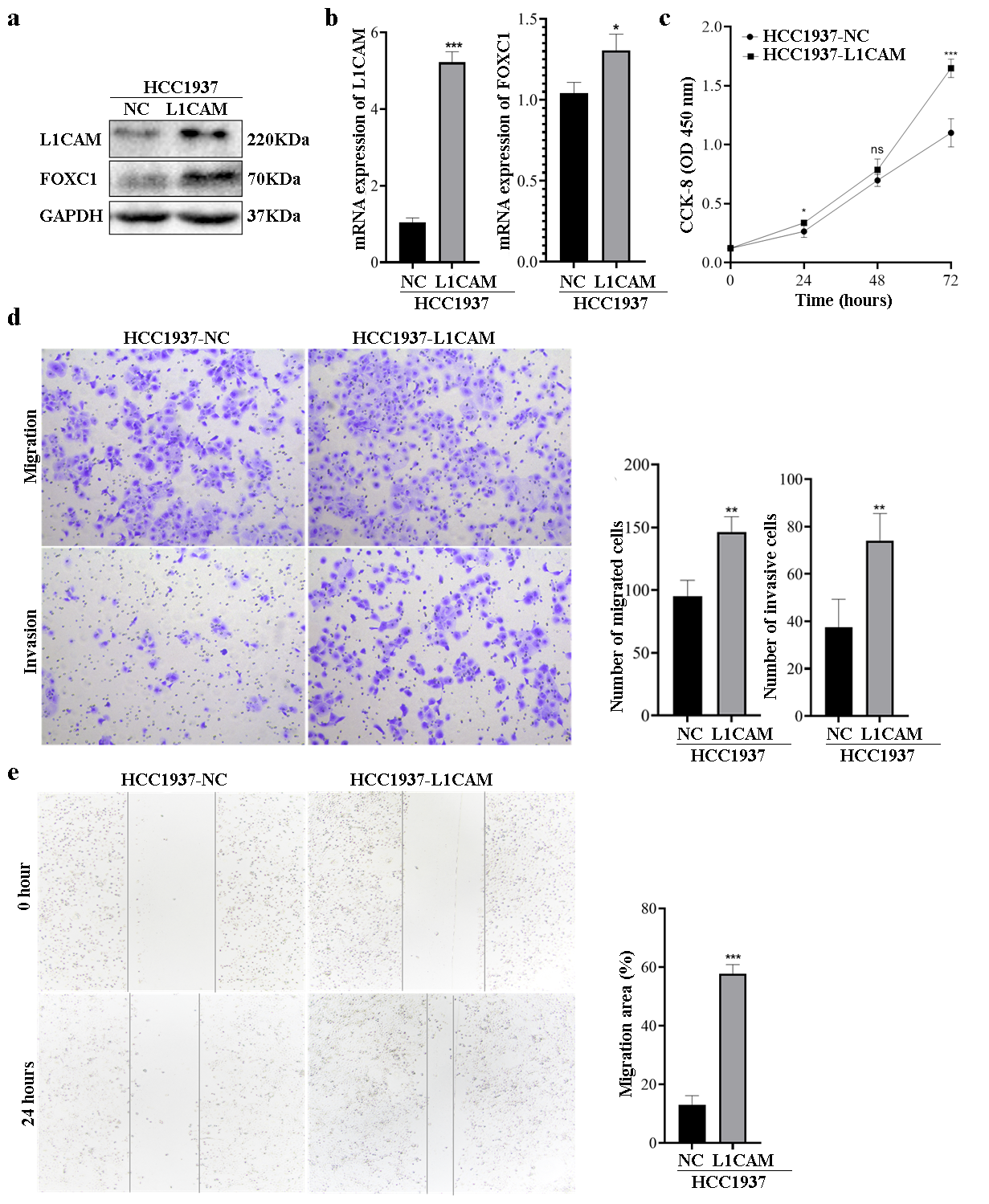

Supplement: Supplementary file 1 — Supplementary file1 (DOC 2680 KB) [file 10549_2023_7177_MOESM1_ESM.doc]
